# Same-day *Enterococcus* qPCR results of recreational water quality at two Toronto beaches provide added public health protection and reduced beach days lost

**DOI:** 10.17269/s41997-023-00763-8

**Published:** 2023-04-17

**Authors:** Faizan Saleem, Herb E. Schellhorn, Albert Simhon, Thomas A. Edge

**Affiliations:** 1 Department of Biology, McMaster University, Hamilton, Ontario Canada; 2 Ontario Ministry of the Environment, Conservation and Parks, Toronto, Ontario Canada

**Keywords:** Beach monitoring, qPCR, Fecal indicator, *E. coli*, *Enterococcus*, Toronto, Surveillance des plages, qPCR, indicateur fécal, *E. coli*, *Enterococcus*, Toronto

## Abstract

**Objectives:**

We evaluated the potential impacts from using a rapid same-day quantitative polymerase chain reaction (qPCR) monitoring method for beach posting outcomes at two Toronto beaches.

**Methods:**

In total, 228 water samples were collected at Marie Curtis Park East and Sunnyside Beaches over the 2021 summer season. Water samples were processed using the USEPA 1609.1 *Enterococcus* qPCR-based method. *Escherichia coli* (*E. coli*) culture data and daily beach posting decisions were obtained from Toronto Public Health.

**Results:**

No significant correlation was observed between previous-day and same-day (retrospective) *E. coli* enumeration results at any Sunnyside Beach transect, and only relatively low (*R* = 0.41–0.56) or no significant correlation was observed at sampling transects for Marie Curtis Park East Beach. Comparing our same-day *Enterococcus* qPCR data to Toronto’s 2-day *E. coli* geometric mean beach posting decisions, we noted the need for additional postings for 1 (2%) and 3 (8%) missed health-risk days at Sunnyside and Marie Curtis Park East Beaches, respectively. The qPCR data also pointed to incorrect postings for 12 (31%) and 6 (16%) lost beach days at Sunnyside and Marie Curtis Park East Beaches, respectively.

**Conclusion:**

Application of a rapid *Enterococcus* qPCR method at two Toronto beaches revealed 5% of beach posting decisions were false negatives that missed health-risk days, while 23% of decisions were false positives resulting in lost beach days. Deployment of the rapid same-day qPCR method offers the potential to reduce both health risks and unnecessary beach postings.

**Supplementary Information:**

The online version contains supplementary material available at 10.17269/s41997-023-00763-8.

## Résumé

**Objectifs:**

Nous avons évalué, à deux plages de Toronto, l’effet possible de l’utilisation d’une méthode de surveillance rapide par PCR quantitative (qPCR) le même jour sur les avis de fermeture ou d’ouverture des plages.

**Méthode:**

En tout, 228 échantillons d’eau ont été prélevés aux plages Marie Curtis Park East et Sunnyside au cours de la saison estivale 2021. La présence d’*Enterococcus* dans les échantillons a été détectée par la méthode USEPA 1609.1, utilisant la qPCR. Les données sur les cultures d’*Escherichia coli (E. coli)* et les avis quotidiens de fermeture ou d’ouverture des plages ont été obtenus auprès du Bureau de santé de Toronto.

**Résultats:**

Aucune corrélation significative n’a été observée entre les résultats (rétrospectifs) du dénombrement de *E. coli* obtenus la veille et le même jour dans les transects de la plage Sunnyside, et une corrélation significative faible (R = 0,41–0,56) ou nulle a été observée dans les transects d’échantillonnage de la plage Marie Curtis Park East. En comparant nos données sur *Enterococcus* obtenues le même jour par qPCR à la moyenne géométrique des avis de fermeture ou d’ouverture des plages sur deux jours liés à *E. coli* émis par le Bureau de santé de Toronto, nous avons remarqué qu’il aurait fallu émettre des avis de fermeture pour 1 jour de risques pour la santé manqué (2 %) à la plage Sunnyside et pour 3 jours de risques pour la santé manqués (8 %) à la plage Marie Curtis Park East. Les données de la qPCR ont aussi fait état d’avis de fermeture incorrects ayant entraîné la perte de 12 jours de plage (31 %) à Sunnyside et de 6 jours de plage (16 %) à Marie Curtis Park East.

**Conclusion:**

L’application d’une méthode de surveillance rapide d’*Enterococcus* par qPCR à deux plages de Toronto a montré que 5 % des avis étaient des faux négatifs qui n’ont pas détecté des jours de risques pour la santé, et que 23 % étaient des faux positifs qui ont entraîné des jours de plage perdus. Le déploiement de la méthode rapide par qPCR le même jour offre la possibilité de réduire à la fois les risques pour la santé et les avis de fermeture de plages inutiles.

## Introduction

Fecal indicator bacteria such as *Escherichia coli* (*E. coli*) and *Enterococcus* are commonly used in North America for water quality monitoring of freshwater and marine beaches, respectively (Health Canada, 2012; USEPA, 2012). Their elevated levels are associated with increased gastrointestinal illnesses in swimmers (USEPA, 2009). Public health authorities monitor beaches for fecal indicator bacteria and make beach posting decisions according to water quality thresholds and beach action values (BAV) defined in recreational water quality criteria and guidelines (USEPA, 2012; Health Canada, 2012). Canadian public health authorities currently rely mainly on culture-based *E. coli* enumeration for freshwater beach monitoring, and beaches are posted to advise against swimming if the geometric mean of *E. coli* colony forming units (CFU) exceeds 200 *E. coli* CFU/100 mL (Health Canada, 2012). However, some jurisdictions (e.g., Toronto Public Health) continue to use a more stringent geometric mean of 100 *E. coli* CFU/100 mL as a beach posting threshold.

Although *E. coli* enumeration by culture is practical and cost-effective for beach monitoring, the results are available at the earliest 18–24 h after sample collection (Haugland et al., 2005, 2016; Siefring et al., 2008). As a result, beach posting decisions are delayed and are at best made using the previous day’s *E. coli* enumeration data. Assumptions regarding the stability of recreational water quality over 24 h can lead to two types of errors: fecal indicator density may have increased above thresholds (false negatives) or decreased below thresholds (false positives). False-negative results can lead to an incorrect assumption that the fecal indicator densities are lower than the beach quality threshold, such that beaches remain open for the public and pose an unrecognized health risk. False-positive results can lead to an incorrect assumption that fecal densities are higher than the beach quality threshold, such that beaches remain posted, and result in an unrecognized lost beach day. Rapid methods such as quantitative polymerase chain reaction (qPCR) that provide same-day results have been shown to provide for valuable same-day decision-making for beach management (Dorevitch et al., 2017).

In 2015, USEPA developed a standard qPCR-based rapid method that can be used for fecal indicator monitoring of recreational water ecosystems (USEPA, 2015). USEPA method 1609.1 is a qPCR-based method for *Enterococcus* quantification in both marine and freshwater. It offers several advantages over culture-based enumeration-based methods. First, the method can provide results within only 3.5–4 h of sample receipt in the lab. Second, beach managers can make timely beach posting decisions using qPCR data on the same day of water sample collection. Third, the National Environmental and Epidemiological Assessment of Recreational water (NEEAR) study has shown that qPCR-based *Enterococcus* quantification measures can be better predictors of gastrointestinal illnesses for freshwater and marine beaches (USEPA, 2009; Wade et al., 2008, 2010). Importantly, *Enterococcus* qPCR-based method 1609.1 also provides minimum standards and rigorous quality control measures to ensure reliability of data for beach posting decision-making. Based on the NEEAR study, the USEPA has established a BAV of ≥ 1000 calibrator cell equivalents (CCE)/100 mL for beach posting decisions using the 1609.1 qPCR-based method. However, USEPA qPCR method 1609.1 for *Enterococcus* has yet to be widely adopted in beach water quality monitoring programs (Shrestha & Dorevitch, 2020).

Despite the growing interest in rapid beach monitoring methods (Health Canada, 2023), and the Canadian Province of Alberta adopting qPCR for *Enterococcus* at beaches (Government of Alberta, 2021), most public health authorities in Canada still rely upon 24-h-old results from culture-based *E. coli* enumeration for beach posting decision-making. The city of Toronto has 10 freshwater beaches along a 42-km Lake Ontario waterfront. Sunnyside and Marie Curtis Park East Beaches are the most impacted by rivers, and they are posted most frequently (often 30–50% of the time in recent years) because of high *E. coli* levels. Toronto Public Health oversees daily water quality testing at all these beaches and makes beach posting decisions using an *E. coli* culture–based enumeration method. As a result, beach posting decisions are delayed about 24 h and are based on previous-day water quality conditions. The city is unique in using a 2-day rolling geometric mean of 100 *E. coli* CFU/100 mL for beach posting decisions. This study builds upon a previous study that provided a validation for applying method 1609.1 at Toronto beaches (Saleem et al., 2022). The primary objectives for our study were (i) testing the correlation between previous-day and same-day (retrospective) *E. coli* enumeration results at two Toronto beaches and (ii) assessing the potential impacts of same-day *Enterococcus* qPCR results compared to Toronto Public Health’s 2-day *E. coli* geometric mean beach posting decisions in 2021.

## Methods

### Water sample collection and filtration processing

**Fig. 1:**
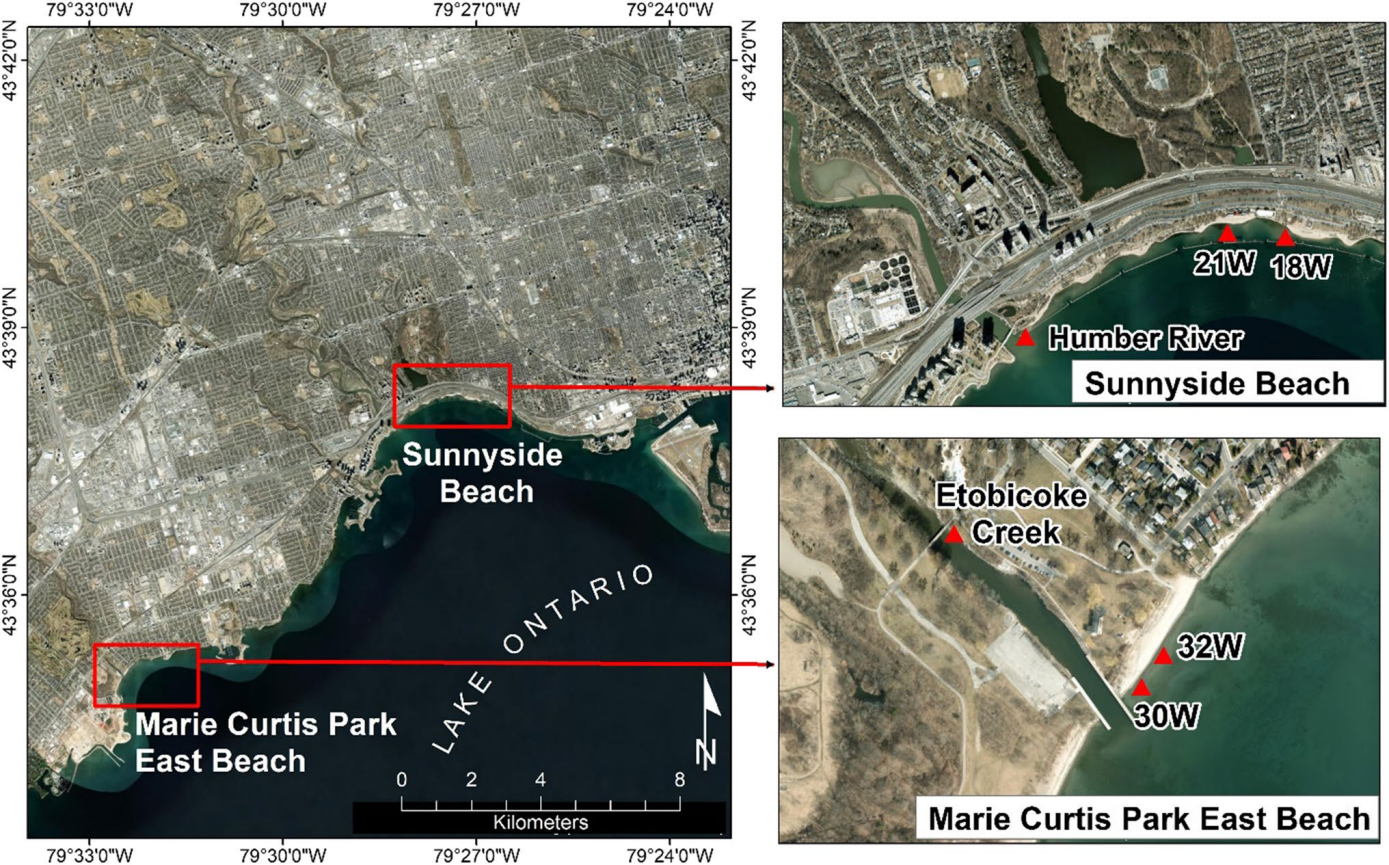
Geographical map of western Toronto, Ontario, and beach sampling sites and water sources (rivers) for Marie Curtis Park East and Sunnyside Beaches

Water samples were collected at chest depth at two freshwater beaches during the summer of 2021. Marie Curtis Park East Beach is located in the west end of Toronto at the mouth of Etobicoke Creek and is exposed to the open waters of Lake Ontario. Sunnyside Beach is also in the west end of Toronto at the mouth of the Humber River, though a breakwall protects it from wave action on Lake Ontario. At each beach, two representative transects were selected from Toronto Public Health’s beach sampling transects that are regularly tested for *E. coli* enumeration by public health authorities (Supplementary Table 1). Water samples were collected on 3 days (two were consecutive days) each week (Mondays, Wednesdays, and Thursdays) over the bathing season such that 228 water samples on 38 beach days were collected from Marie Curtis Park East Beach (30W and 32W transects, Fig. 1 and Supplementary Table 1) and Sunnyside Beach (18W and 21W transects, Fig. 1 and Supplementary Table 1). Grab water samples were collected just below the water surface in 1 L sterile screw-capped polyethylene terephthalate (PET) bottles. Water samples were collected between about 5:30 a.m. and 7:00 a.m., stored on ice, and transported to the laboratory by 8:00 a.m. Upon receipt, water samples were processed by USEPA Method 1609.1 for *Enterococcus* qPCR (USEPA, 2015). Briefly, 100 mL (0.1 L) of water sample was passed through a 0.45 µm polycarbonate membrane filter (Millipore Corp., Bedford, MA) for collection of bacterial biomass. The membrane filter was subsequently bead beaten with extraction buffer (0.2 µg/mL Salmon sperm DNA in acetate-EDTA buffer pH 9) and centrifuged at 12,000 g for 1 min, followed by collection of 400 µL supernatant. Collected supernatant was centrifuged again at 12,000 g for 5 min, and 350 µL of DNA extract was collected for qPCR.

### qPCR quality controls

A standard curve was prepared following USEPA 1609.1 by performing tenfold dilutions of DNA extracted from 10^9^ *Enterococcus faecalis* CFUs (ATCC 29212). The DNA standard with the highest concentration comprised 40,000 target sequence copies (TSC)/5 µL, while the DNA standard with the lowest TSC represented the lowest limit of standard curve quantification (LLOSQ = 10 TSC/5 µL). A calibrator positive control for qPCR was prepared by diluting *E. faecalis* stock culture with sterile phosphate buffer saline (pH: 7.5) to 10^4^ *Enterococcus faecalis* CFUs. The phosphate buffer saline matrix spike was prepared by dispensing 1000 µL of calibrator positive control solution into 100 mL buffer. The water reference matrix spike for each sampling site included 100 mL of water sample spiked with positive calibrator control. The non-template control included a qPCR mixture with 5 µL of sterile water instead of sample DNA. A more detailed description of quality controls can also be accessed in Saleem et al. (2022).

### qPCR mixture and amplification protocol

USEPA 1609.1 method was used for the qPCR analysis of *Enterococcus* quantification in water samples (USEPA, 2015). The reaction mixture (25 µL) included 12.5 µL of TaqMan Environmental Master mix (TaqMan™), 5 µL of template DNA, 2.5 µL of bovine serum albumin (2.0 mg/mL), 3.0 µL of primer/probe working solution (final concentration of primers and probe was 1.0 µM and 80.0 nm, respectively), and 2.0 µL of sterile nuclease-free water. The primer/probe working solution was prepared by diluting stock solutions (primer stock: 500 µM and probe stock: 100 µM) with sterile nuclease-free water. Each qPCR run comprised eight water samples, positive calibrator controls, and non-template controls, and phosphate buffer saline matrix spike was used once a week for ongoing precision recovery analysis. qPCRs were carried out in 96-well qPCR plates (Corning Inc., USA) on a Bio-Rad CFX96 Touch Real-Time PCR Detection System thermocycler (Bio-Rad Inc., USA). A detailed description of the qPCR protocol can be accessed in a previous publication (Saleem et al., 2022).

### Data analysis

Daily culture-based *E. coli* enumeration data (Province of Ontario standard membrane filtration method) from June 1 to August 31, 2021, were kindly shared by Toronto Public Health for all sampling transects at Sunnyside and Marie Curtis Park East Beaches. Data received from the public health authority also included the information about which beach days were posted for summer 2021. USEPA 1609.1 *Enterococcus* qPCR protocol quantifies the ratio of DNA target sequence from calibrator positive control and water samples which normalizes for differences in DNA recovery using sample processing control (Salmon sperm DNA; spiked in samples before DNA extraction) (Haugland et al., 2016; USEPA, 2015). All the qPCR calculations were performed using the standard Excel sheet provided by USEPA (https://www.epa.gov/sites/default/files/2015-08/methods_1609-1-1611-1-calculation-spreadsheet-april-2015.xlsx). *E. coli* enumeration and *Enterococcus* qPCR data were log_10_-transformed. Toronto Public Health’s culture-based *E. coli* enumeration data were cleaned up by removal of data points lower than or equal to the lower limit of quantification (10 CFU/100 mL) and were used for correlation testing between their previous-day (results from the first day in two consecutive days of sample collection) and same-day (results from the second day in two consecutive days of sample collection) *E. coli* results. It should be recognized that same-day culture-based *E. coli* data was only possible from a retrospective perspective as it required a 24-h incubation delay. All data were tested for normal distribution by using Shapiro–Wilk’s for normality testing (*p* > 0.05), followed by Pearson correlation analysis with a *p* value cutoff ≤ 0.05 at 95% confidence interval (http://www.sthda.com/english/wiki/correlation-test-between-two-variables-in-r). GGpubr (ggscatter) (Kassambara & Kassambara, 2020) and GGplot2 (Wickham et al., 2016) packages were used for the construction of correlation plots, followed by adjustment of *p* values using p.adjust function in R (Jafari & Ansari-Pour, 2019).

## Results

### qPCR quality analysis

In total, 228 water samples were collected, corresponding to 38 beach days for summer 2021. Method 1609.1 met all standards and quality control criteria, and Table 1 describes the qPCR parameters for method quality assessment. All the beach samples passed quality parameters for good DNA recovery (sample processing control < 3 Ct difference in comparison to calibrator positive controls) and qPCR inhibition (internal amplification control within 1.5 Ct difference in comparison to non-template control).

**Table 1:** Data quality measures for quality control analysis for qPCR

Data quality parameters		
Number of standard curves	4	Standard curves quality analysis
*R*^2^ (mean ± standard deviation)	0.998 ± 0.003	
Number of calibrators	36	Calibrator positive controls quality analysis
*Enterococci* qPCR threshold cycle value (mean ± standard deviation)	31.3 ± 0.7	
Internal amplification control (IAC) qPCR threshold cycle value (mean ± standard deviation)	31.2 ± 0.5	
Sample processing control qPCR threshold cycle value (mean ± standard deviation)	21.5 ± 0.9	
Number of samples	**228**	Beach water samples quality analysis
*Enterococci* qPCR threshold cycle value (mean ± standard deviation)	34.8 ± 2.3	
Internal amplification control qPCR threshold cycle value (mean ± standard deviation)	31.1 ± 0.6	
Sample processing control (SPC) qPCR threshold cycle value (mean ± standard deviation)	22.2 ± 0.3	
qPCR inhibition (IAC Ct > 1.5)	0	Beach water samples qPCR analysis
Poor DNA recovery (SPC Ct > 3)	0	

### Correlation between previous-day and retrospective same-day *E. coli* enumeration

**Fig. 2:**
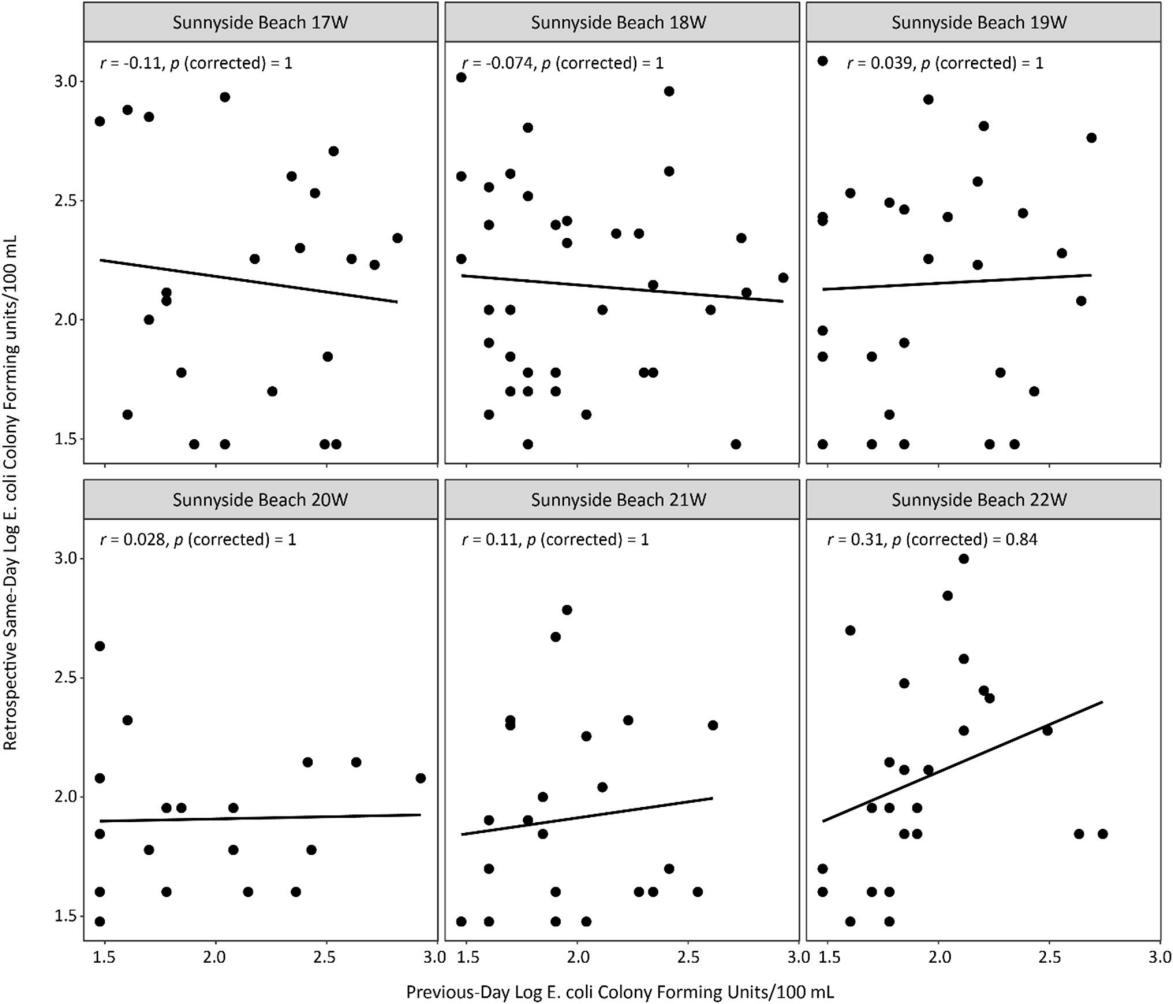
Correlation between previous-day and retrospective same-day log *E. coli* CFU/100 mL for Sunnyside Beach sampling transects. Correlation analysis was performed by using Pearson correlation at 95% confidence interval. *E. coli* data were obtained from Toronto Public Health for analysis

**Fig. 3:**
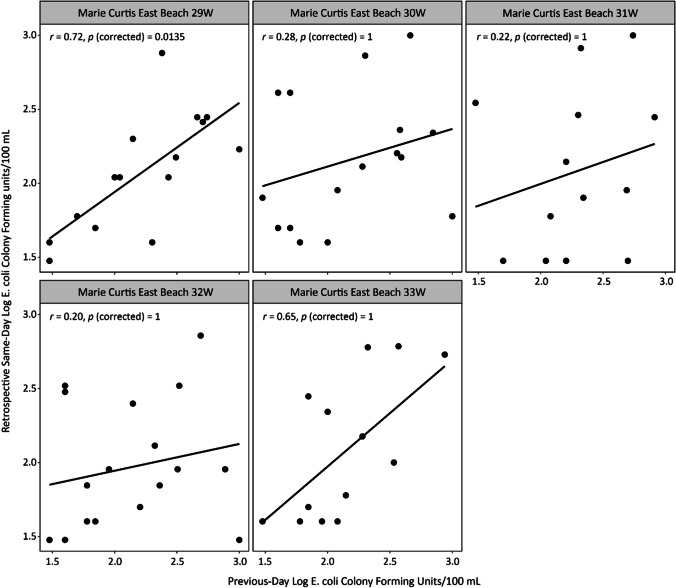
Correlation between previous-day and retrospective same-day log *E. coli* CFU/100 mL for Marie Curtis Park East Beach sampling transects. Correlation analysis was performed by using Pearson correlation at 95% confidence interval. *E. coli* data were obtained from Toronto Public Health for analysis

*E. coli* culture–based enumeration results obtained from Toronto Public Health for summer 2021 were used to test the correlation between previous- and same-day *E. coli* results. Figures 2 and 3 represent the correlation scatter plots for Sunnyside and Marie Curtis Park East Beaches, respectively. While correlations were better at Marie Curtis Park East Beach, perhaps due to its closer proximity to the river mouth and more immediate mixing unrestrained by a breakwall, nonetheless, there were notably weak correlations at both beaches. All six sampling transects of Sunnyside Beach, and one transect at Marie Curtis Park East Beach, revealed no significant correlation between previous-day and retrospective same-day *E. coli* results (*p* > 0.05). Four of five sampling transects (29W, 30W, 31W, and 33W) at Marie Curtis Park East Beach presented only low to moderate correlation (*R* = 0.41–0.56, *p* ≤ 0.05) between previous-day and same-day *E. coli* results.

### Comparison of same-day (retrospective) and 2-day rolling *E. coli* geometric mean for beach postings outcomes

An analysis of Toronto Public Health’s *E. coli* data comparing beach posting outcomes from same-day data (retrospective) and the city’s actual posting decisions based on their 2-day geomean revealed quite different results for beach postings if Toronto Public Health could have obtained, hypothetically, same-day culture-based *E. coli* data (Supplementary Fig. 1 and 2). The city’s own *E. coli* results indicated that Marie Curtis Park East Beach and Sunnyside Beach were either incorrectly posted or kept open 40% and 30% of the time, respectively, over the 2021 bathing season. In particular, same-day *E. coli* results would have indicated that Marie Curtis Park East Beach and Sunnyside Beach should have been posted on an extra 17 and 15 days, respectively, in 2021, which were not indicated by 2-day geometric *E. coli* data.

### Impact of same-day qPCR-based monitoring on beach posting outcomes

**Fig. 4:**
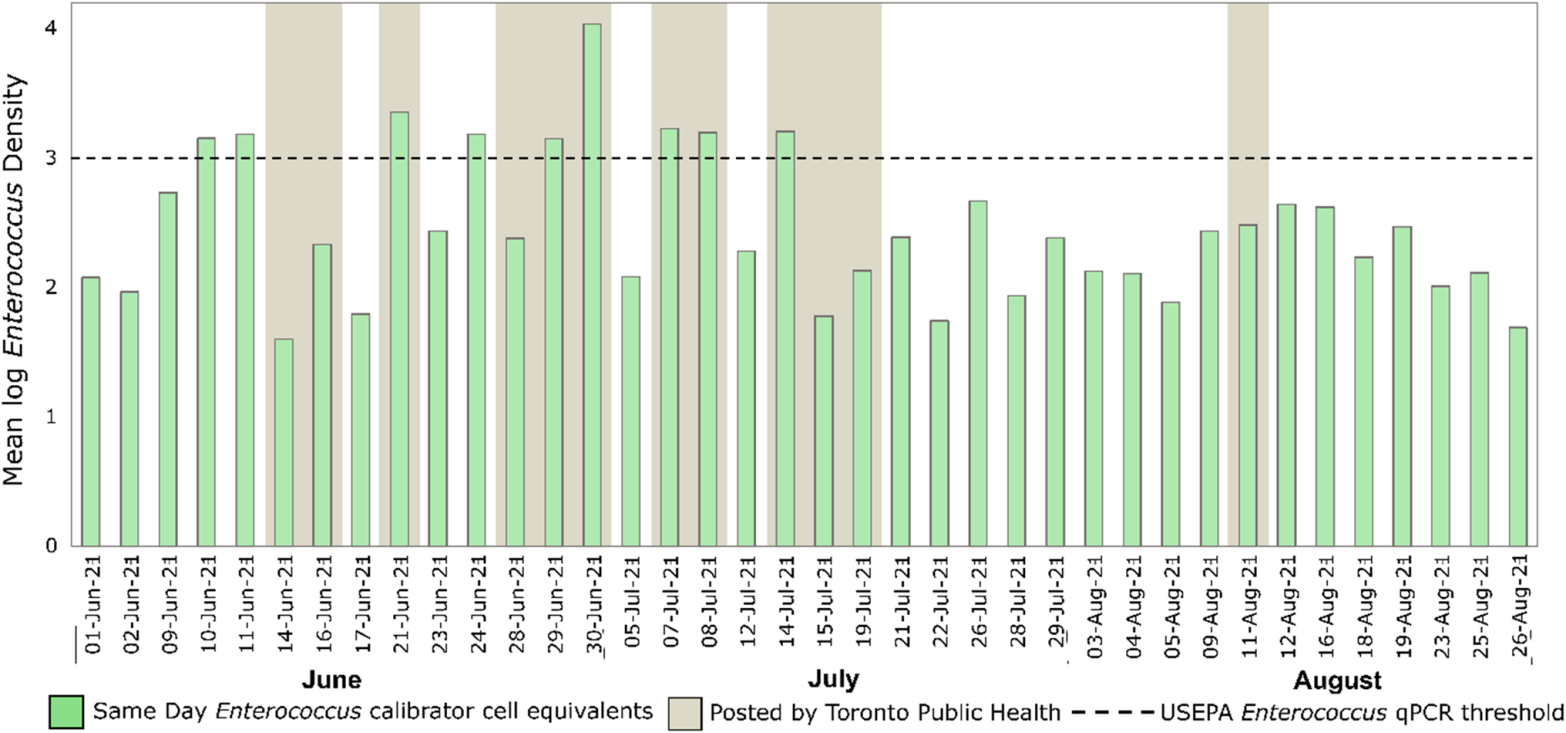
Comparison between Toronto Public Health’s beach posting decisions and same-day *Enterococcus* qPCR quantification for Marie Curtis Park East Beach. Dashed line shows USEPA beach action value for *Enterococcus* quantification by qPCR (≥ 1000 CCE/100 mL), while gray highlighted days represent beach days posted by Toronto Public Health based on their rolling 2-day *E. coli* geometric mean

**Fig. 5:**
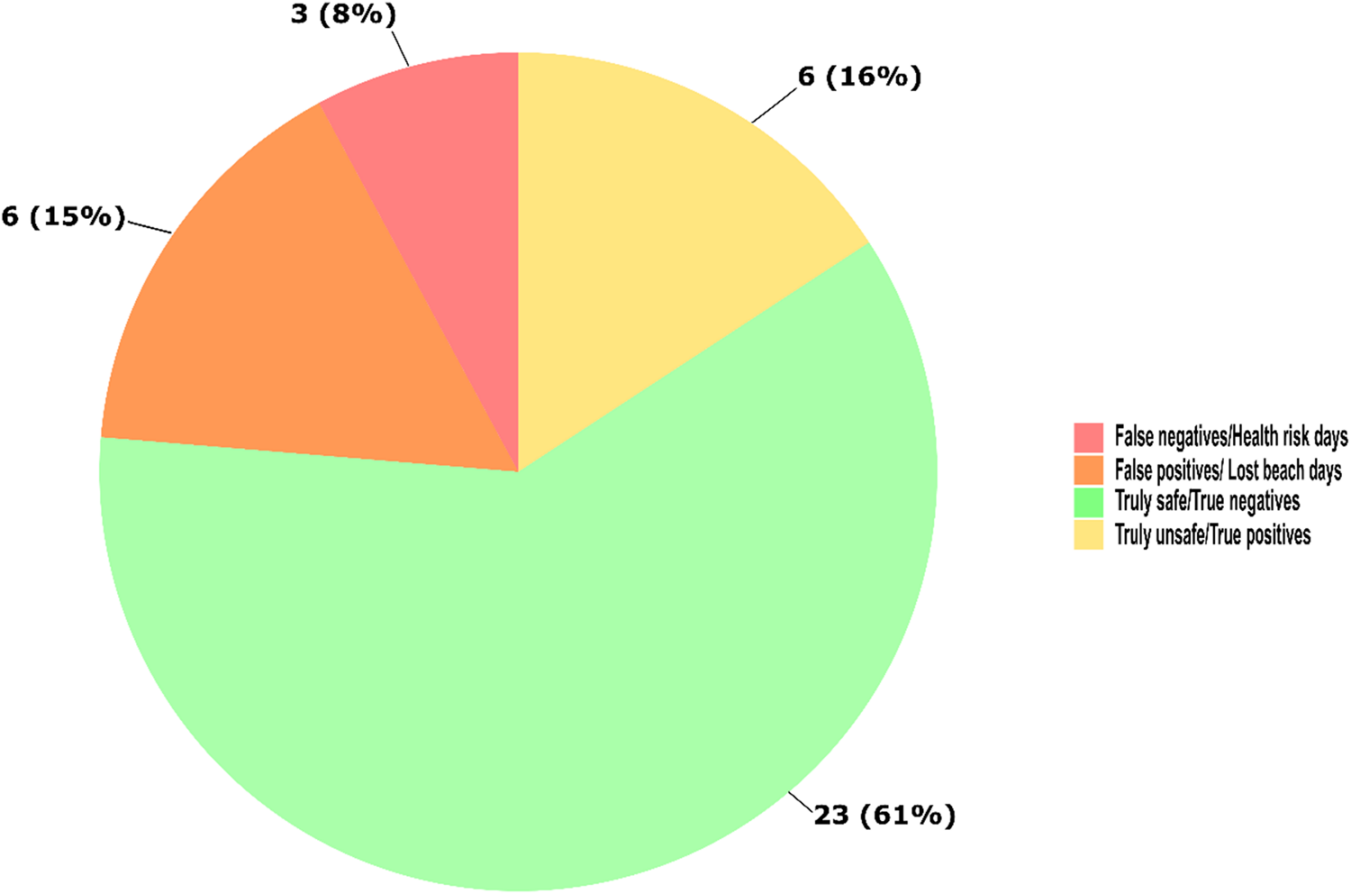
Analysis of impacts on Marie Curtis Park East Beach posting decision-making using the *Enterococcus* qPCR and compared to Toronto Public Health’s reported 2-day rolling *E. coli* geometric mean for summer 2021 beach days (*n* = 38)

Considerable differences were observed in beach posting outcomes when qPCR results were compared to Toronto Public Health’s 2-day rolling *E. coli* geometric mean. Beach posting differences were categorized as false negatives (when Toronto Public Health did not post the beach on the basis of their 2-day rolling geometric mean for culturable *E. coli* data, but same-day *Enterococcus* qPCR indicated the need for a beach posting) and false positives (when Toronto Public Health posted the beach on the basis of their 2-day rolling geometric mean for culturable *E. coli* data, but same-day *Enterococcus* qPCR indicated the beach could have remained open). For Marie Curtis Park East Beach (Fig. 4), 12 out of 38 tested beach days (32%) were posted according to Toronto Public Health’s 2-day rolling *E. coli* geometric mean, while our same-day qPCR-based *Enterococcus* indicated only 9 beach days (24%) when the beach should have been posted. Of the 38 tested beach days (Fig. 5), our same-day qPCR data identified 3 false-negative/health-risk days (8%) and 6 false-positive/lost beach days (16%).

**Fig. 6:**
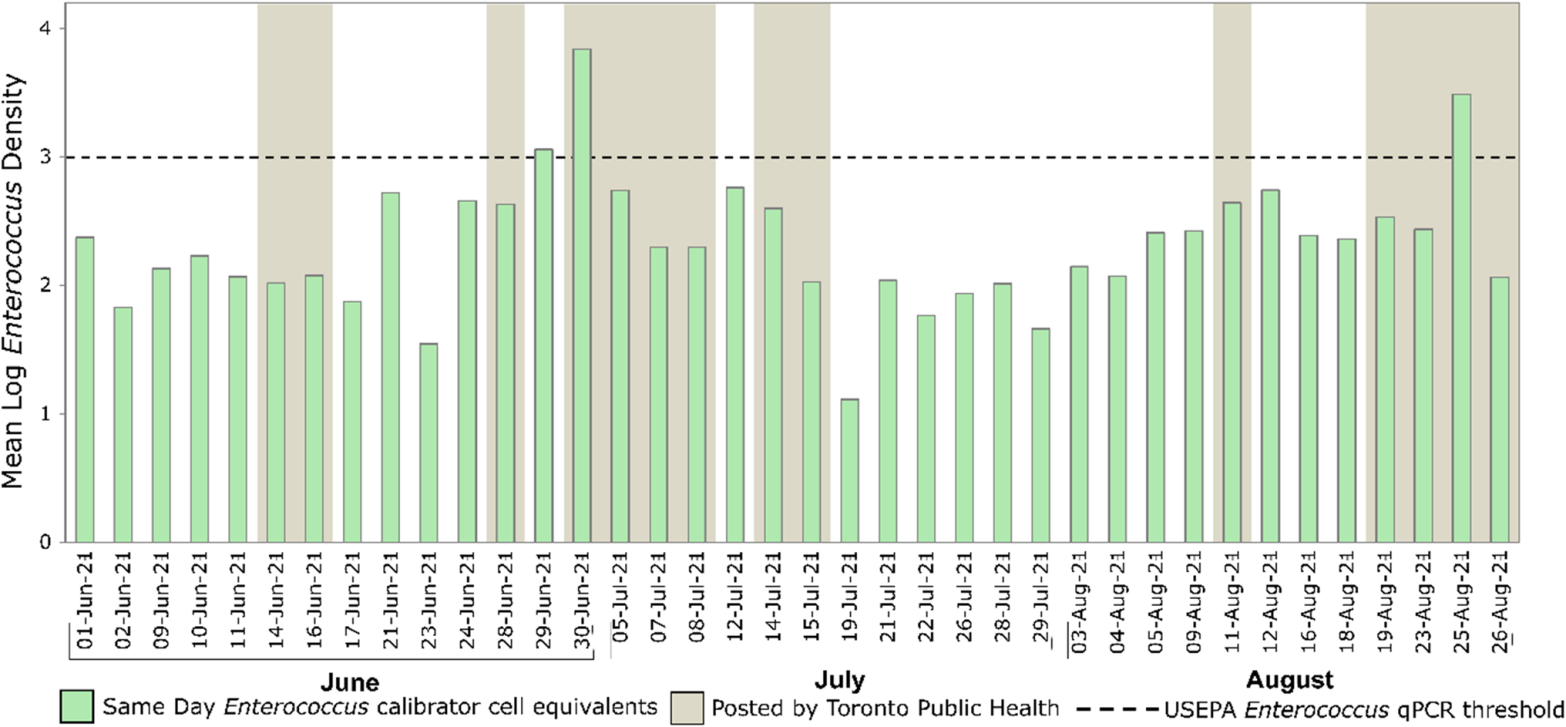
Comparison between Toronto Public Health’s beach posting decisions and same-day *Enterococcus* qPCR quantification for Sunnyside Beach. Dashed line shows USEPA beach action value for *Enterococcus* quantification by qPCR (≥ 1000 CCE/100 mL), while gray highlighted days represent beach days posted by Toronto Public Health based on its rolling 2-day *E. coli* geometric mean

**Fig. 7:**
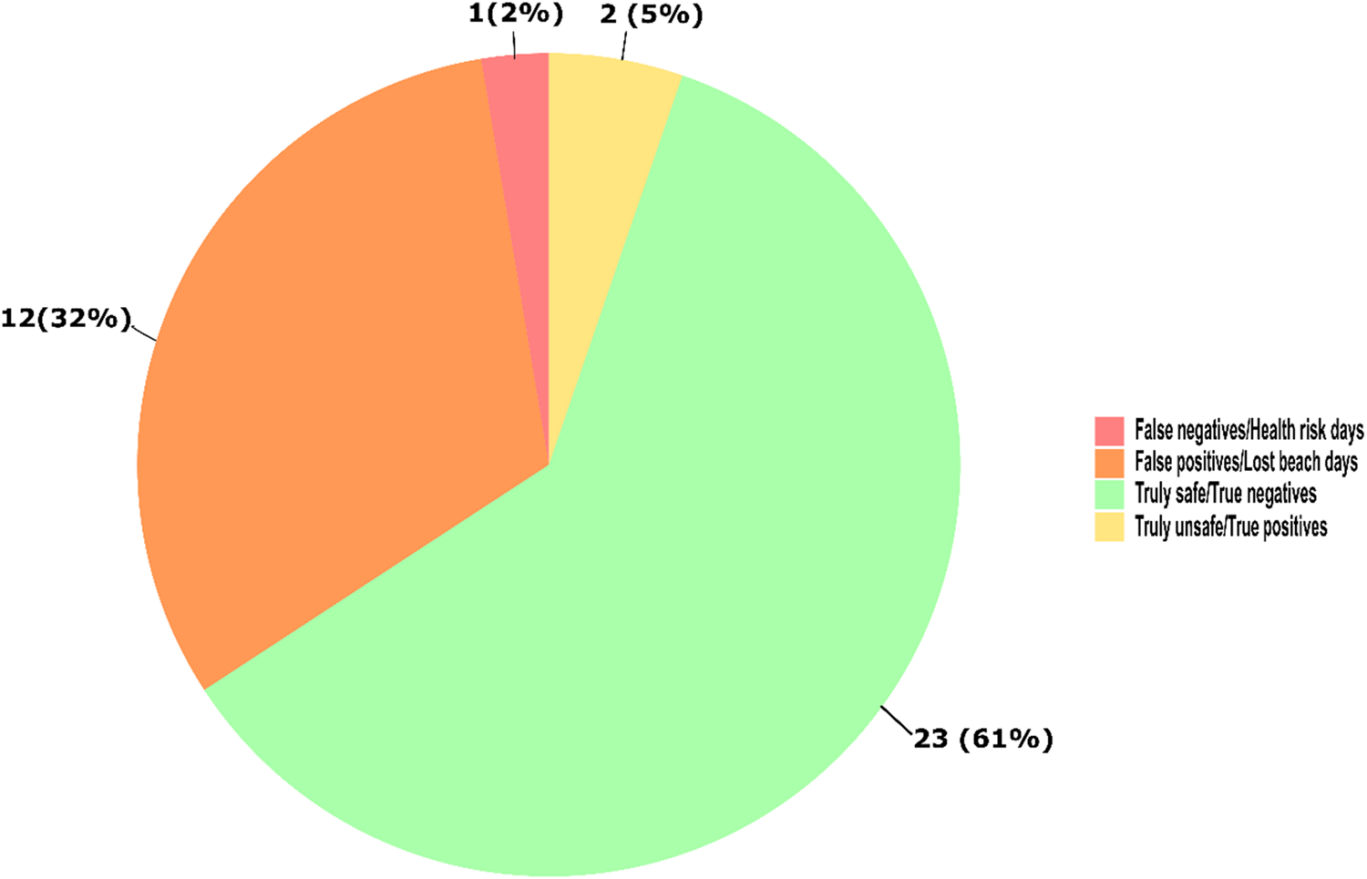
Analysis of impacts on Sunnyside Beach posting decision-making using the *Enterococcus* qPCR and compared to Toronto Public Health’s reported 2-day rolling *E. coli* geometric mean for summer 2021 beach days (*n* = 38)

For Sunnyside Beach (Fig. 6), 14 out of 38 tested beach days (37%) were posted according to Toronto Public Health’s 2-day rolling *E. coli* geometric mean, while our same-day qPCR-based *Enterococcus* quantification indicated only 3 beach days when the beach should have been posted. Of the 38 tested beach days (Fig. 7), our same-day qPCR data identified 1 false-negative/health-risk day (2%) and 12 false-positive/lost beach days (31%).

Using our same-day qPCR results for beach monitoring would have resulted in a gain of 6 and 12 beach open days for Marie Curtis Park East and Sunnyside Beaches, respectively, on our sampling days. Additionally, qPCR monitoring would have prevented 3 and 1 health-risk days for Marie Curtis Park East and Sunnyside Beaches, respectively, on our sampling days.

## Discussion

Microbial monitoring methodologies for freshwater recreational sites are being revised as new more robust measures can provide more timely results (Campbell & Kleinheinz, 2020). Our study provides a comparison of beach posting outcomes between Toronto’s culture-based *E. coli* method and our same-day *Enterococcus* qPCR method for Marie Curtis Park East and Sunnyside Beaches. In total, 228 water samples corresponding to 38 beach days were collected over the summer 2021 season. Water samples were collected each day by 7:00 a.m., transported within 1 h to the lab, and processed in the lab within 3.5–4 h of sample receipt. qPCR quantification results were typically provided to Toronto Public Health by noon on a given day. Our experience suggests that this qPCR approach can work for beaches located within about a 1-h drive from a qPCR lab, provided that the water sampling is completed by about 7:00 a.m. A practical limitation will be the flexibility to have water samples collected earlier in the morning than typically now done.

Toronto Public Health’s monitoring program uses a 2-day rolling *E. coli* geometric mean for beach posting decision-making. However, *E. coli* counts are subject to change in beaches during a 24-h delay (Enns et al., 2012; Whitman et al., 1999, 2004). Daily *E. coli* enumeration data from Toronto were obtained from June 1 to August 31, 2021, to assess the correlation between previous-day and same-day (retrospective) *E. coli* enumeration results. Even the city of Toronto’s own *E. coli* data showed no significant correlation was observed between the previous- and same-day *E. coli* results at most beach sampling transects, and only relatively low correlations were observed at some Marie Curtis Park East Beach transects. Incorporating another earlier day to calculate the city’s 2-day geometric mean would likely even further reduce any potential for correlation with same-day (retrospective) *E. coli* results. In addition, our analysis of previous-day and same-day (retrospective) *E. coli* results often resulted in different beach posting outcomes. These findings are consistent with two previous studies on US beaches that reported significant changes in fecal indicator bacteria numbers over a time scale of hours (Converse et al., 2012; Dorevitch et al., 2017). Additionally, it was observed that for Chicago beaches, previous-day *E. coli* results had no better than a 50% chance at correctly predicting next-day’s water quality (Dorevitch et al., 2017). Therefore, reliance upon 24-h-old *E. coli* results may not provide a true representation of beach water quality and can result in many erroneous beach postings.

The qPCR-based monitoring method (USEPA 1609.1) is designed to overcome the time limitation of culture-based enumeration methods and can lead to more rapid beach posting decision-making for beach managers. Based on our qPCR-based results, *Enterococcus* qPCR quantification would have allowed the Toronto beaches to remain open for additional days. Similarly, studies done on Chicago beaches identified that a culture-based *E. coli* method generated three times more beach postings in comparison to *Enterococcus* qPCR (Dorevitch et al., 2017). However, studies on Wisconsin beaches have found that *Enterococcus* qPCR generated higher numbers of beach exceedances in comparison to *E. coli/Enterococcus* defined substrate medium–based culturing methods (Campbell & Kleinheinz, 2020; Sheth et al., 2016). These results suggest the impacts from applying the *Enterococcus* qPCR method may differ between beaches necessitating site-specific evaluations and understanding prior to adoption. Differences in qPCR method validation and outcomes between the geographical locations can be due to different fecal contamination sources/fecal indicators, water chemistry interfering with PCR, environmental parameters (rainfall, wave height), or the levels of non-viable cells detectable by qPCR (Lavender and Kinzelman, 2009; Telech et al., 2009). Before implementing the *Enterococcus* qPCR method, comparative testing with existing/conventional beach monitoring methods (e.g., *E. coli* culture–based methods) should be performed to determine the level of agreement between the methods for new beach settings (Sheth et al., 2016).

This study found substantial differences in beach posting outcomes using our same-day qPCR method compared to the city’s previous 2-day *E. coli* geometric mean data even though our study was limited to two beaches and only 38 beach days for the 2021 summer season. These differences might be partially explained by our two beaches being located near river mouths and more rapidly impacted by fluctuating river water quality that a 24-h-delayed culture-based method may not detect on some days as fast as a same-day qPCR method. The difference in Toronto Public Health beach posting outcomes using a rapid qPCR method could also be because Toronto Public Health beach posting decisions are based on a 2-day rolling *E. coli* geometric mean that incorporates culture-based *E. coli* results from 2 days before a posting decision, in addition to previous-day results. Our results provide guidance for future studies to investigate implications of adopting more rapid qPCR testing methods for beaches. Our results indicate rapid qPCR methods can enable more timely beach posting decisions to better protect public health and reduce adverse social, tourism, and economic impacts from incorrect postings.

## Conclusions

1. USEPA Method 1609.1 for *Enterococcus* by qPCR provided same-day results, within 5.5 to 6 h of recreational water sample collection and within 3.5 to 4 h of sample receipt in the laboratory, and it can provide a more rapid beach monitoring approach for beach managers to avoid erroneous beach postings based on 24-h-old *E. coli* data.
2. In comparison to the city of Toronto’s 2-day rolling *E. coli* geometric mean data over 38 tested beach days, our same-day qPCR data identified 12 false-positive/lost beach days at Sunnyside Beach and 6 false-positive/lost beach days at Marie Curtis Park East Beach.
3. In comparison to the city of Toronto’s 2-day rolling *E. coli* geometric mean data over 38 tested beach days, our same-day qPCR data identified 3 false-negative/health risk days at Marie Curtis Park East Beach and 1 false-negative/health risk day at Sunnyside Beach.
4. Use of the same-day *Enterococcus* qPCR method would probably contribute to many more differences in beach posting decisions if applied every day for the complete summer season across all 10 Toronto beaches.

## Contributions to knowledge

What does this study add to existing knowledge?

- This study presents the *Enterococcus* qPCR-based beach monitoring method as a potential alternative to traditional culture-based enumeration methods being used at freshwater beaches in Canada. It shows that by providing faster same-day results, the *Enterococcus* qPCR can reduce the number of erroneous beach postings currently associated with using 24-h-delayed culture-based testing.

What are the key implications for public health interventions, practice, or policy?

- The qPCR-based method provides results within 3.5–4 h of sample receipt and can allow beach managers to provide more timely and reliable beach posting decisions to protect public health by reducing the number of unrecognized health risk days, and by reducing adverse social and economic impacts from incorrect postings and unrecognized lost beach days.

## Supplementary Information

Below is the link to the electronic supplementary material.

Supplementary file1 (DOCX 188 KB)

## Data Availability

Data are available upon request.
