# Supplementary material for: Same-day Enterococcus qPCR results of recreational water quality at two Toronto beaches provide added public health protection and reduced beach days lost
Source: Can J Public Health. 2023 Apr 17;114(4):676–87. doi: 10.17269/s41997-023-00763-8 (PMC10349029; doi:10.17269/s41997-023-00763-8)
Supplement: Supplementary file 1 — Supplementary file1 (DOCX 188 KB) [file 41997_2023_763_MOESM1_ESM.docx]

Canadian Journal of Public Health

Title: Same-day *Enterococcus* qPCR Results of Recreational Water Quality at Two Toronto Beaches Provide Added Public Health Protection and Reduced Beach Days Lost

**Supplementary Information**

**Supplementary Table 1.** Geographical coordinates of sampling sites for both Marie Curtis Park East and Sunnyside Beaches

| Beach | Sampling Site (Transect) | Latitude | Longitude |
| --- | --- | --- | --- |
| Marie Curtis Park East Beach | 30W | 43.585610 | -79.540054 |
|  | 32W | 43.585110 | -79.540560 |
| Sunnyside Beach | 18W | 43.636612 | -79.452670 |
|  | 21W | 43.637110 | -79.457530 |


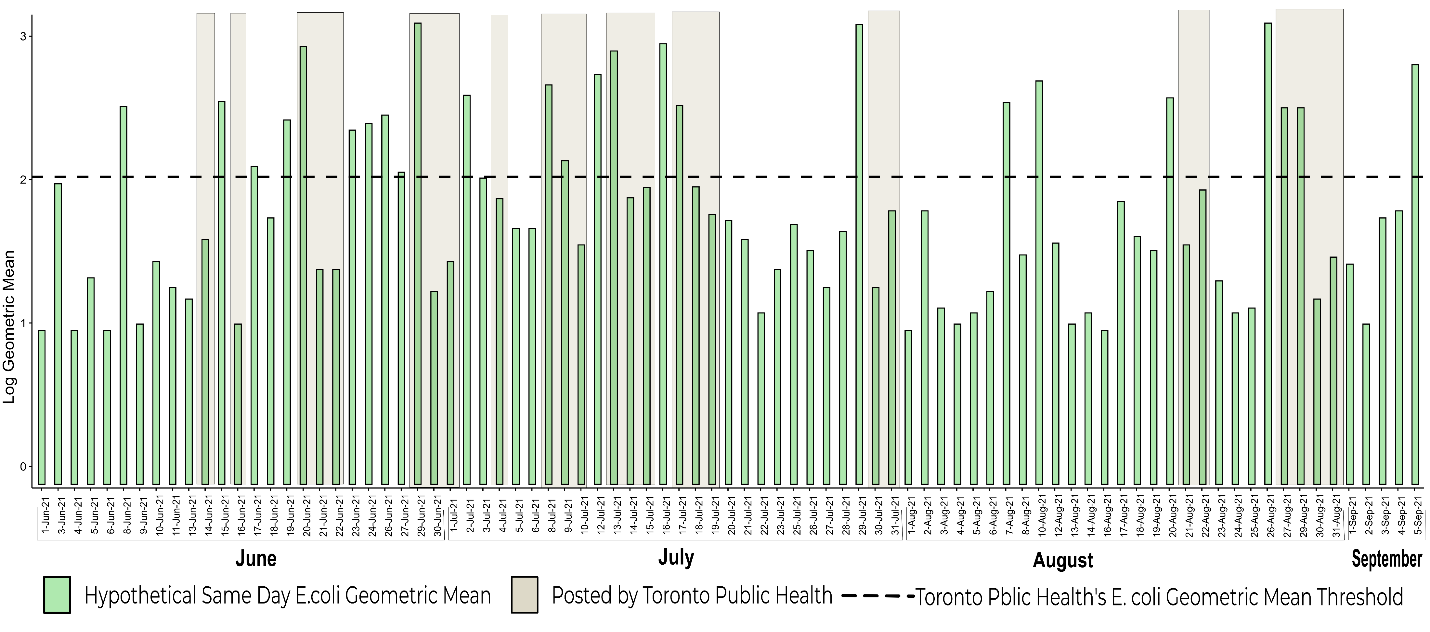


Supplementary Figure 1. Difference in Beach Postings for Marie Curtis Park East Beach according to Toronto Public Health’s data for summer 2021, if hypothetically, *E. coli* culturing results were available on the same day of sample collection.


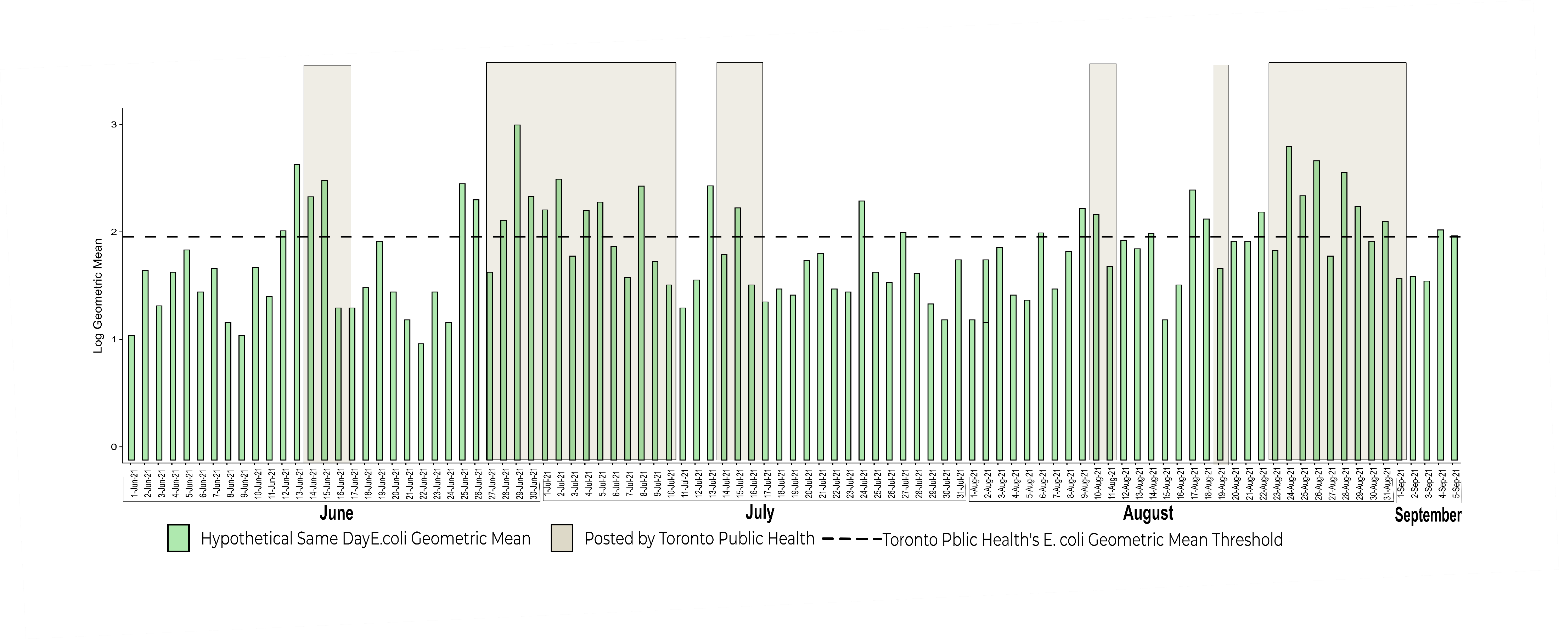


Supplementary Figure 2. Difference in Beach Postings for Sunnyside Beach according to Toronto Public Health’s data for summer 2021, if hypothetically, *E. coli* culturing results were available on the same day of sample collection.
